# 

**Published:** 1893-02-11

**Authors:** 


PRICE TWOPENCE.

No. 333, Vol. XIII.—Feb. 11th, 1893.

[Registered at the General Post Office as a Newspaper for transmission in the United Kingdom and Abroad.]

WITH EXTRA NURSING SUPPLEME[NT]
Per Annum, 8s. 6d., post free.
Monthly Parts, 6d.; post free, 8d.
Ditto per Annum, 8s., post free.

